# Reviewer acknowledgement 2013

**DOI:** 10.1186/1742-4690-11-5

**Published:** 2014-02-06

**Authors:** Andrew Lever, Mark Wainberg

**Affiliations:** 1 University of Cambridge, Cambridge, UK; 2 McGill University AIDS Centre and Jewish General Hospital, Montreal, Canada

## Abstract

**Contributing reviewers:**

*Retrovirology* would like to sincerely thank the following for giving their time and expertise to review manuscripts for the journal in 2013. Their support for the journal is greatly appreciated.

## 

Truus Abbink

United Kingdom

Catherine Adamson

United Kingdom

Marylyn M. Addo

United States of America

Philippe Vicente Afonso

France

Sunil Ahuja

United States of America

Christopher Aiken

United States of America

Hirofumi Akari

Japan

John Albin

United States of America

Jose Alcami

Spain

Ghalib Alkhatib

United States of America

Neil Almond

United Kingdom

Marcus Altfeld

United States of America

Zandrea Ambrose

United States of America

Emma Anderson

United Kingdom

Takashi Angata

Taiwan

Nathalie Arhel

France

James Arthos

United States of America

Eric Arts

United States of America

Eran Bacharach

Israel

Jonathan Ball

United Kingdom

Charles Bangham

United Kingdom

Norbert Bannert

Germany

Matjaz Barboric

Finland

Francis Barin

France

Andreas Baur

Germany

Ali Bazarbachi

Lebanon

Karen Beemon

United States of America

Brendan Bell

Canada

Serge Benichou

France

Monsef Benkirane

France

Yamina Bennasser

France

Ben Berkhout

Netherlands

Clarisse Berlioz-Torrent

France

Neil Berry

United Kingdom

Francoise Bex

Belgium

James Binley

United States of America

Fabien Blanchet

France

Joel Blankson

United States of America

Jonas Blomberg

Sweden

Robert Blumenthal

United States of America

Morgane Bomsel

France

Kathleen Boris-Lawrie

United States of America

Persephone Borrow

United Kingdom

Fadila Bouamr

United States of America

Simon Brackenridge

United Kingdom

Martine Braibant

France

Denys Brand

France

Bernard Branson

United States of America

Bluma Brenner

Canada

Mark Brockman

Canada

James Bruce

United States of America

Shilpa Buch

United States of America

Michael Bukrinsky

United States of America

Mary Jo Burkhard

United States of America

John Burnett

United States of America

Frederic Bushman

United States of America

Paul U Cameron

Australia

Edward Campbell

United States of America

Timothy Cardozo

United States of America

Jeremy Carlton

United Kingdom

William Carr

United States of America

Rob Center

Australia

Anna Cereseto

Italy

Benjamin Chen

United States of America

Bing Chen

United States of America

Peter Cherepanov

United Kingdom

Frauke Christ

Belgium

Claudia Cicala

United States of America

Andrea Cimarelli

France

Angela Ciuffi

Switzerland

Paul Clapham

United States of America

Janice Clements

United States of America

Charles Cobbs

United States of America

Alan Cochrane

Canada

John Coffin

United States of America

Eric Cohen

Canada

Myron Cohen

United States of America

Mark Connors

United States of America

Robert Craigie

United States of America

Martin Cranage

United Kingdom

Gael Cristofari

France

Bryan Cullen

United States of America

Atze Das

Netherlands

Siddhartha Datta

United States of America

D A Davis

United States of America

Paul de Bakker

Netherlands

Hugues de Rocquigny

France

Steven Deeks

United States of America

Joachim Denner

Germany

Cynthia Derdeyn

United States of America

Benjamin Descours

France

Francesca Di Nunzio

France

Felipe Diaz-Griffero

United States of America

Gilles Divita

France

Aaron Donahue

Canada

Tao Dong

United Kingdom

Margherita Doria

Italy

Lucy Dorrell

United Kingdom

Ivan D'Orso

United States of America

Daniel Douek

United States of America

Joseph Dougherty

United States of America

John Elder

United States of America

Michael Emerman

United States of America

Stephane Emiliani

France

Alan Engelman

United States of America

Jerome Estaquier

Canada

Eliseo Eugenin

United States of America

Oliver Fackler

Germany

Hung Fan

United States of America

Ariberto Fassati

United Kingdom

Benoit Favier

France

Eberhard Fiebig

United States of America

Adam Fletcher

United Kingdom

Dan Frampton

United Kingdom

Pierre Frange

France

Eric Freed

United States of America

Jun-ichi Fujisawa

Japan

Axel Fun

Netherlands

Hansjakob Furrer

Switzerland

Vitaly Ganusov

United States of America

Feng Gao

United States of America

Guangxia Gao

China

J. Victor Garcia

United States of America

Gerardo Garcia-Lerma

United States of America

Jeremy Garson

United Kingdom

Anne Gatignol

Canada

Jonathan Geiger

United States of America

Anna Maria Geretti

United Kingdom

Denis Gerlier

France

Antoine Gessain

France

Mauro Giacca

Italy

Nicolas Gilbert

France

M John Gill

Canada

Amber Gillett

Australia

Stephen Goff

United States of America

Maureen Goodenow

United States of America

Martin Goodier

United Kingdom

Robert J Gorelick

United States of America

Paul Gorry

Australia

Matthias Gotte

Canada

Heinrich Gottlinger

United States of America

Caroline Goujon

United Kingdom

Thomas Gramberg

Germany

Jane Greatorex

United Kingdom

Patrick Green

United States of America

Dirk Grimm

Germany

Harriet Groom

United Kingdom

Elisabetta Groppelli

United Kingdom

John Gross

United States of America

John Guatelli

United States of America

Huldrych Guenthard

Switzerland

Naveed Gulzar

Canada

Samuel Gunderson

United States of America

Ju-Tao Guo

United States of America

Ravindra Kumar Gupta

United Kingdom

Hillel Haim

United States of America

Lou Hammarskjold

United States of America

Yingshan Han

Canada

Gordon Harkiss

United Kingdom

David Harrich

Australia

Mark Harris

United Kingdom

Reuben Harris

United States of America

Ronald Harty

United States of America

Alex Harwig

Netherlands

Jonathan Heeney

United Kingdom

Joshua Herbeck

United States of America

Andrew Hill

Australia

Vanessa Hirsch

United States of America

Amnon Hizi

Israel

Carol Hlela

South Africa

Africa Holguin

Spain

Thomas Hope

United States of America

Margaret Hosie

United Kingdom

Peter Hraber

United States of America

Maite Huarte

Spain

Amy Hulme

United States of America

Eric Hunter

United States of America

Gero Hutter

Germany

Tatsuhiko Igarashi

Japan

Yukihito Ishizaka

Japan

Dmitri Ivanov

United States of America

Richard Jackson

United Kingdom

Martin Roelsgaard Jakobsen

Denmark

Leo James

United Kingdom

Kuan-Teh Jeang

United States of America

Rienk Jeeninga

Netherlands

Richard Jenner

United Kingdom

Shibo Jiang

China

Dong-Yan Jin

Hong Kong

Marc Johnson

United States of America

Clare Jolly

United Kingdom

Albert Jordan

Spain

Sarah Joseph

United States of America

Anjali Joshi

United States of America

David Kabat

United States of America

Taku Kambayashi

United States of America

Jonathan Karn

United States of America

Fatah Kashanchi

United States of America

George Kassiotis

United Kingdom

David Katzenstein

United States of America

Daniel Kaufmann

Canada

Norihito Kawashita

Japan

Michael Kay

United States of America

Mary Kearney

United States of America

Anthony Kelleher

Australia

Stephen Kent

Australia

Julia Kenyon

United Kingdom

Frank Kirchhoff

Germany

Scott Kitchen

United States of America

Zachary Klase

United States of America

P J Klasse

United States of America

Renate Koenig

Germany

Neeltje Kootstra

Netherlands

Roger Kouyos

Switzerland

Christine Kozak

United States of America

Hans-Georg Kraeusslich

Germany

Victor Kramer

Canada

Mamuka Kvaratskhelia

United States of America

Martin R B Löchelt

Germany

Aksana Labokha

United Kingdom

Nadine Laguette

France

Philipp Lang

Germany

Lewis Lanier

United States of America

Roger Le Grand

France

Christine Leib-Mösch

Germany

Jonathan Leis

United States of America

David N Levy

United States of America

Ronald Levy

United States of America

Sharon Lewin

Australia

Chen Liang

Canada

Mathias Lichterfeld

United States of America

Judy Lieberman

United States of America

Florian Liegeois

Cameroon

Ying-Ju Lin

Taiwan

Min Lu

United States of America

Jeremy Luban

United States of America

Paul Luciw

United States of America

Vladimir V Lukashov

Netherlands

Paolo Lusso

United States of America

Gary Maartens

South Africa

Renaud Mahieux

France

Johnson Mak

Australia

Frank Maldarelli

United States of America

Fabrizio Mammano

France

Nicolas Manel

France

Louis Mansky

United States of America

Laura Manuelidis

United States of America

Alessandro Marcello

Italy

David Margolis

United States of America

Florence Margottin-Goguet

France

David Markovitz

United States of America

Susan Marriott

United States of America

Mark Marsh

United Kingdom

Fabiola Martin

United Kingdom

Cynthia Masison

United States of America

Antonio Mastino

Italy

Masao Matsuoka

Japan

Wendy Maury

United States of America

Domenico Mavilio

Italy

Myra McClure

United Kingdom

Mark McNally

United States of America

Gregory Melikyan

United States of America

Zora Melkova

Czech Republic

Luis Menendez-Arias

Spain

Jean-Michel Mesnard

France

Thibault Mesplede

Canada

A Dusty Miller

United States of America

Mahesh Mohan

United States of America

Hoi Ping Mok

United Kingdom

Debasis Mondal

United States of America

Eugenio Montini

Italy

Penny Moore

South Africa

Lynn Morris

South Africa

Donald Mosier

United States of America

Walther Mothes

United States of America

Andrew J Mouland

Canada

Barbara Mueller

Germany

Viktor Müller

Hungary

Carsten Münk

Germany

Delphine Muriaux

France

Edward L Murphy

United States of America

Matteo Negroni

France

Stuart Neil

United Kingdom

Sergei Nekhai

United States of America

Mark Nelson

United Kingdom

Hironori Nishitsuji

Japan

Philip Norris

United States of America

Mahdad Noursadeghi

United Kingdom

Vlad Novitsky

United States of America

Jeremy Nuttall

United States of America

David O’Connor

United States of America

Una O’Doherty

United States of America

Akihiko Okayama

Japan

Akira Ono

United States of America

Marcel Ooms

United States of America

Dominique Ouellet

Canada

Julie Overbaugh

United States of America

Annette Oxenius

Switzerland

Mirko Paiardini

United States of America

Jean-Christophe Paillart

France

Paul Palumbo

United States of America

Gianfranco Pancino

France

Ivona Pandrea

United States of America

Dimitrios Paraskevis

Greece

Vincent Parissi

France

Jo-Ann Passmore

South Africa

Alexander Pasternak

Netherlands

Vinay Pathak

United States of America

William Paxton

Netherlands

Finn Skou Pedersen

Denmark

Martine Peeters

France

Danielle Perez Bercoff

Luxembourg

Lucía Pérez-Álvarez

Spain

Sallie Permar

United States of America

Fred Perrino

United States of America

Boris Matija Peterlin

United States of America

Louis Picker

United States of America

Vincent Piguet

United Kingdom

Sabine Piller

Australia

Abraham Pinter

United States of America

Massimo Pizzato

Italy

JC Plantier

France

Stefan Pöhlmann

Germany

Georgios Pollakis

Netherlands

Larisa Poluektova

United States of America

Art Poon

Canada

Pantelis Poumbourios

Australia

Christopher Power

Canada

Vinayaka Prasad

United States of America

Damian Purcell

Australia

Yudong Quan

Canada

Bharat Ramratnam

United States of America

R Keith Reeves

United States of America

Jan Rehwinkel

United Kingdom

Alan Rein

United States of America

Axel Rethwilm

Germany

Andrew Rice

United States of America

Laura Richert-Spuhler

United States of America

Marjorie Robert-Guroff

United States of America

Harriet Robinson

United States of America

Michael Roche

Australia

Olivier Rohr

France

Morgane Rolland

United States of America

Nicola Rose

United Kingdom

Susan Ross

United States of America

Monica Roth

United States of America

Kenneth Roux

United States of America

Sarah Rowland-Jones

United Kingdom

Christopher Rowley

United States of America

Walter Royal

United States of America

Jamil Saad

United States of America

Jonah Sacha

United States of America

Anthony Sadler

Australia

Asier Saez-Cirion

France

Manish Sagar

United States of America

Mineki Saito

Japan

Kalle Saksela

Finland

Nitin Saksena

Australia

Jesus Salazar-Gonzalez

United States of America

Marco Salemi

United States of America

Johan Sandberg

Sweden

Rogier Sanders

Netherlands

Mario Santiago

United States of America

Stefanos Sarafianos

United States of America

Quentin Sattentau

United Kingdom

Daniel Sauter

Germany

Gabriella Scarlatti

Italy

Susan Schader

United States of America

Michael Schindler

Germany

Olivier Schwartz

France

Daniel Scott-Algara

France

Nabila Seddiki

France

Oliver Semmes

United States of America

Barbara Shacklett

United States of America

Robert Shafer

United States of America

George Shaw

United States of America

Keisuke Shindo

Japan

Robert Siliciano

United States of America

John Sinclair

United Kingdom

Jacek Skowronski

United States of America

Richard David Sloan

United Kingdom

Nicolas Sluis-Cremer

United States of America

Redmond Smyth

France

Marcelo Soares

Brazil

Joseph Sparano

United States of America

Paul Spearman

United States of America

Roberto Speck

Switzerland

Anna-Lena Spetz

Sweden

Tzanko Stantchev

United States of America

Laura Steel

United States of America

Heribert Stoiber

Austria

Jonathan Stoye

United Kingdom

Klaus Strebel

United States of America

Peter Sun

United States of America

Valentina Svicher

Italy

Ronald Swanstrom

United States of America

Gilda Tachedjian

Australia

Akifumi Takaori-Kondo

Japan

Yasuhiro Takeuchi

United Kingdom

Frédéric Tangy

France

Jamal Tazi

France

Amalio Telenti

Switzerland

John Tilton

United States of America

Bruce Torbett

United States of America

Greg Towers

United Kingdom

Jozsef Tozser

Hungary

Mary-Anne Trabaud

France

Michel J Tremblay

Canada

Alexandra Trkola

Switzerland

Athe Tsibris

United States of America

Stuart Turville

Australia

Derya Unutmaz

United States of America

Tonja van der Kuyl

Netherlands

Carine Van Lint

Belgium

Koen Van Rompay

United States of America

Anne-Mieke Vandamme

Bermuda

Guido Vanham

Belgium

Bruno Verhasselt

Belgium

Eric Vivier

France

Volker M Vogt

United States of America

Annapurna Vyakarnam

United Kingdom

Stephen Waggoner

United States of America

Chin-Tien Wang

Taiwan

Jonathan Weber

United Kingdom

Robin Weiss

United Kingdom

Sarah Welbourn

United States of America

Raymond Welsh

United States of America

James Whitney

United States of America

Brian Wigdahl

United States of America

Brian Willett

United Kingdom

Bryan Williams

Australia

Mark Wills

United Kingdom

Kenneth Witwer

United States of America

Joseph Wong

United States of America

Li Wu

United States of America

Yuntao Wu

United States of America

Yoshihisa Yamano

Jordan

Masahiro Yamashita

United States of America

Melvyn Yap

United Kingdom

Venkat Yedavalli

United States of America

Osamu Yoshie

Japan

Jerome Zack

United States of America

Alessia Zamborlini

France

John Zaunders

Australia

Steven Zeichner

United States of America

Richard Y Zhao

United States of America

Yong-Hui Zheng

United States of America

Michael Zwick

United States of America

